# The global burden of liver disease: a challenge for methods and for public health

**DOI:** 10.1186/s12916-014-0159-5

**Published:** 2014-09-18

**Authors:** Peter Byass

**Affiliations:** WHO Collaborating Centre for Verbal Autopsy, Epidemiology and Global Health, Department of Public Health and Clinical Medicine, Umeå University, Umeå, 90187 Sweden; Medical Research Council/Wits University Rural Public Health and Health Transitions Research Unit (Agincourt), School of Public Health, Faculty of Health Sciences, University of the Witwatersrand, Johannesburg, South Africa; Institute of Applied Health Sciences, School of Medicine and Dentistry, University of Aberdeen, Aberdeen, UK

**Keywords:** Liver disease, Cirrhosis, Mortality, Verbal autopsy, Alcohol consumption, Hepatitis, Global estimates, Vaccination, Risk factors, Civil registration

## Abstract

New Global Burden of Disease estimates for liver cirrhosis, published in *BMC Medicine*, suggest that cirrhosis caused over a million deaths in 2010, with a further million due to liver cancer and acute hepatitis. Cause-specific mortality data were very sparse for some regions, particularly in Africa, with no relevant mortality data for 58/187 countries. Liver disease involves infectious, malignant and chronic aetiologies with overlapping symptoms. Where available mortality data come from verbal autopsies, separating different types of liver disease is challenging.

Cirrhosis is a disease of rich and poor alike; key public health risk factors such as alcohol consumption play an important role. Risk-reduction strategies such as controlling the price of alcohol are being widely discussed. Since these estimates used alcohol consumption as a covariate, they cannot be used to explore relationships between alcohol consumption and cirrhosis mortality.

There is hope: coming generations of adults will have been vaccinated against hepatitis B, and this is envisaged to reduce the burden of fatal liver disease. But more complete civil registration globally is needed to fully understand the burden of liver disease.

Please see related article: http://www.biomedcentral.com/1741-7015/12/145/abstract.

## Background

Cirrhosis is late-stage liver disease, in which the liver develops scarring as a result of various long-term challenges. Precipitating causes of cirrhosis include viral hepatitis and heavy alcohol consumption, and it is associated with risks for developing primary hepatocellular carcinoma. Advanced cirrhosis is a life-threatening condition with limited treatment options.

In a new assessment of liver cirrhosis mortality from the Global Burden of Disease (GBD) project, published in *BMC Medicine*, a diversity of methods and data sources led to a global estimate of just over one million deaths in 2010, which was approximately 2% of all deaths [1]. Alongside this, GBD estimates suggest a further million deaths were due to liver cancer and acute hepatitis. Overall, therefore, liver disease represents a considerable public health burden.

If every death from liver disease across the globe were investigated and documented in an efficient, standardised manner, we could proceed with much greater clarity into the realm of public health strategies for reducing this global burden. Unfortunately that is far from the case, and so the sophisticated modelling techniques that GBD has brought to bear on measuring cirrhosis and other liver disease are important. Nevertheless, mathematical models of disease patterns work best when fed with large quantities of high quality data; great care has to be taken not to over-extend modelling assumptions in trying to span the considerable holes in whatever data may be available [2].

## What do the estimates show?

**Figure 1:**
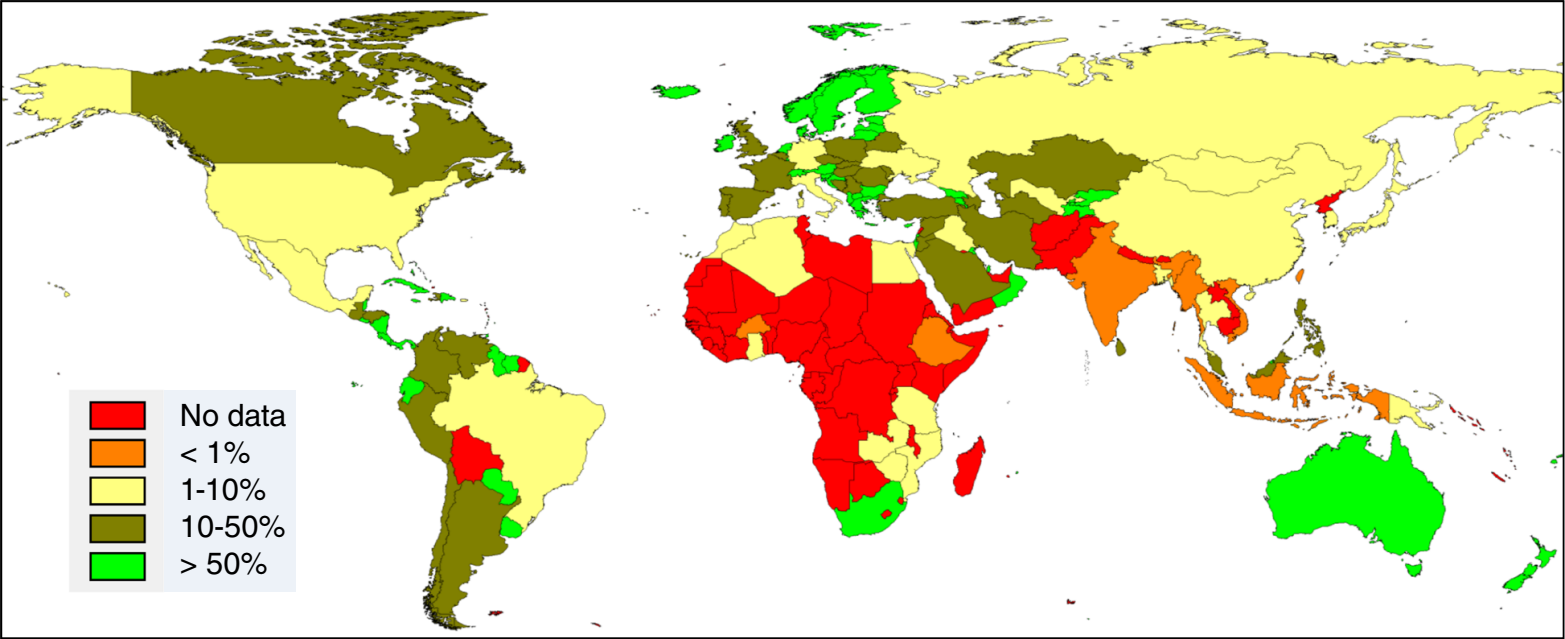
**Ratio of observations used in the Global Burden of Disease model to the number of cirrhosis deaths estimated for 2010, by country.**

As presented in the GBD study, the paucity of mortality data on cirrhosis, particularly in sub-Saharan Africa, is striking. For the overall 96,639 observations of cirrhosis mortality for 1980-2010 used in the GBD model, Figure 1 shows the ratio of observations used to the number of cirrhosis deaths estimated for 2010, by country. For 58/187 (31%) of countries there were no observations. Countries where observations numbered less than 1% of estimated deaths accounted for 39% of the world’s population. In sub-Saharan Africa, excluding South Africa, over 90% of observations came from verbal autopsy sources, mostly originating from INDEPTH (International Network for the Demographic Evaluation of Populations and their Health) Network sites [3], rather than from national vital registration data. The GBD team appropriately notes that their estimates for sub-Saharan Africa must be treated cautiously.

Liver disease, of which cirrhosis is an important component, encompasses infectious, malignant and chronic disease processes arising from a wide range of aetiologies, which also have some key symptomatology in common (such as jaundice and abdominal distension). Accordingly it becomes a cross-cutting concept in terms of cause of death classifications [4]. This is turn brings methodological challenges when diagnostic outcomes are unclear or incomplete. Verbal autopsy (where, for otherwise undocumented deaths, a post-mortem interview is carried out with relatives or witnesses of the death to ascertain circumstances and symptoms, which can then be interpreted into a likely cause of death) is the main source of cause of death data where deaths are not routinely certified, as is the case for much of Africa and Asia. For verbal autopsy procedures in particular, assigning a cause of death with effective discrimination between acute infectious hepatitis, liver cancer or cirrhosis is likely to be challenging, unless the interview respondent has a clear sense of a clinical diagnosis. In the WHO 2012 verbal autopsy standard [5], liver cirrhosis is included as cause of death category VA06.02, covering ICD-10 codes K70-K76, but it is also likely in reality that some less well-defined cases of cirrhosis may fall into VA06.01 (acute abdomen) or VA98 (other and unspecified non-communicable disease). In addition, there is a distinct possibility of misclassification under VA02.02 (digestive neoplasms).

At the same time, it must be remembered that cirrhosis is a disease of the rich as well as the poor. Every continent on the GBD mortality map for 2010 includes countries with cirrhosis mortality estimates both above 30 per 100,000 and below 10 per 100,000. If all 187 countries are ranked by estimated cirrhosis mortality rates for 2010, then the USA, Germany and Portugal are all examples from the mid-tertile, alongside others from Latin America, Africa and Asia. This reflects the complex mixture of risk factors and disease processes involved.

Alcohol consumption undoubtedly plays an important role in the development of cirrhosis, cutting across geographic, political and economic boundaries. A national study from Iceland has shown significant increases in cirrhosis mortality as state-controlled restrictions on alcohol marketing have relaxed [6]. A modelling study of non-communicable disease risk-reduction strategies has suggested that reducing alcohol consumption (as one of six risk factors) could contribute substantially to avoiding large numbers of premature deaths worldwide [7]. Public health strategies to achieve reductions in alcohol consumption at the population level are also a matter of considerable debate; it has been suggested that controlling the minimum price of units of alcohol would specifically target heavy drinkers, and thus those at greatest risk of cirrhosis [8]. However, the GBD estimates of cirrhosis mortality need careful interpretation in relation to alcohol consumption as a public health issue. Alcohol consumption estimates were used as a covariate in the GBD modelling, and, particularly for males, emerged as a highly ranked component of the ensemble model. Therefore one must presume that alcohol consumption strongly drove the mortality estimates, particularly for countries with limited mortality data, bringing in an element of circularity between alcohol consumption and cirrhosis mortality. Thus the GBD results should not be used to explore relationships between alcohol consumption (or other covariates) and cirrhosis mortality.

## Signs of hope

From working in West Africa in the 1980s, one poignant memory is of young adult males with severe liver disease who were obviously present in almost every hospital ward. Before AIDS-related mortality became common, liver disease was well known as a major killer of young, productive adults in that region. The majority of today’s adults were born before hepatitis B vaccination was widely implemented, but there are signs of hope that, in decades to come, fatal adult liver disease as a consequence of widespread hepatitis B infection in infancy will decrease due to vaccination. The GBD estimates suggest that hepatitis B accounted for a particularly high population attributable fraction of cirrhosis mortality in sub-Saharan Africa (though noting that estimates of chronic hepatitis B infection were included as a model covariate). At least it should be possible to fill important gaps in the evidence base on using hepatitis B vaccination for reducing fatal liver disease as the Gambia Hepatitis Intervention Study cohort moves on into high-risk age groups [9].

## Conclusion

The new GBD estimates of cirrhosis mortality are undoubtedly interesting and important. However, they carry the major uncertainties associated with all such modelling. They must therefore be regarded as an interim solution, while the world, and Africa in particular, moves towards much more effective and widespread civil registration systems to fill the data gaps [10].
